# Immunohistochemical characterization of the M4 macrophage population in leprosy skin lesions

**DOI:** 10.1186/s12879-018-3478-x

**Published:** 2018-11-15

**Authors:** Jorge Rodrigues de Sousa, Francisco Dias Lucena Neto, Mirian Nacagami Sotto, Juarez Antonio Simões Quaresma

**Affiliations:** 1 Instituto Evandro Chagas, Secretaria de Vigilância em Saúde, Ministério da Saúde, Ananindeua, PA Brazil; 2 Núcleo de Medicina Tropical, Universidade Federal do Pará, Belém, PA Brazil; 3 Centro de Ciências Biológicas e da Saúde, Universidade do Estado do Pará, Belém, PA Brazil; 4 Faculdade de Medicina, Universidade de São Paulo, São Paulo, SP Brazil; 5 Instituto de Medicina Tropical de São Paulo, Universidade de São Paulo, São Paulo, SP Brazil; 6 Núcleo de Medicina Tropical, UFPA, Av. Generalíssimo Deodoro 92, Umarizal, Belém, Pará 66055-190 Brazil

**Keywords:** Macrophage, Immunohistochemistry, Mycobacteria, Immunology

## Abstract

**Background:**

Since macrophages are one of the major cell types involved in the *Mycobacterium leprae* immune response, roles of the M1 and M2 macrophage subpopulations have been well defined. However, the role of M4 macrophages in leprosy or other infectious diseases caused by mycobacteria has not yet been clearly characterized. This study aimed to investigate the presence and potential role of M4 macrophages in the immunopathology of leprosy.

**Methods:**

We analyzed the presence of M4 macrophage markers (CD68, MRP8, MMP7, IL-6, and TNF-α) in 33 leprosy skin lesion samples from 18 patients with tuberculoid leprosy and 15 with lepromatous leprosy by immunohistochemistry.

**Results:**

The M4 phenotype was more strongly expressed in patients with the lepromatous form of the disease, indicating that this subpopulation is less effective in the elimination of the bacillus and consequently is associated with the evolution to one of the multibacillary clinical forms of infection.

**Conclusion:**

M4 macrophages are one of the cell types involved in the microbial response to *M. leprae* and probably are less effective in controlling bacillus replication, contributing to the evolution to the lepromatous form of the disease.

## Background

Leprosy is a chronic infectious disease caused by *Mycobacterium leprae*, an obligate intracellular bacillus that infects macrophages, dendritic cells, and Schwann cells [1, 2]. Leprosy is considered a neglected disease that represents a serious public health problem in developing countries [3, 4].

Clinically, leprosy shows spectral behavior in which the clinical evolution of the disease and associated histopathological changes are dependent on the host immune response. According to the Ridley-Jopling classification based on clinical, histopathological, immunological, and bacilloscopic criteria, leprosy presents in five main clinical forms: tuberculoid leprosy (TT), borderline-tuberculoid leprosy (BT), borderline-borderline leprosy (BB), borderline-lepromatous leprosy (BL), and lepromatous leprosy (LL) [5, 6].

The clinical evolution of the disease is closely related with the immune response triggered in the host. Given the spectral nature of the disease, with well-defined clinical and immunological presentations at each stage, leprosy represents an efficient model for investigating the host–parasite relationship [7, 8]. In the TT form, the cellular response is mediated by T helper (Th)1 lymphocytes, which produce cytokines that induce a pro-inflammatory response. In the LL form, the cellular immune response is characterized by the predominance of Th2 lymphocytes, which trigger a suppressive response. In the forms BT, BB, and BL, the cellular response presents a heterogeneous differentiation pattern that varies between the cellular responses in the TT and LL forms [1, 7, 8].

Previous studies have shown that according to the evolution or chronicity of spectral diseases, certain cell groups show a response that polarizes between pro- and anti-inflammatory activities. In this context, macrophages belong to a group of cells associated with the innate immune response that undergo phenotypic modification and produce receptors, co-stimulatory molecules, enzymes, and cytokines that induce the development of the suppressive or inflammatory response [9–11].

In the TT form of leprosy, activation of the classical pathway by M1 macrophages induces the production of tumor necrosis factor-alpha (TNF-α), interferon-gamma (IFN-γ), and induced nitric oxide synthase (iNOS), which induce the generation of free radicals that destroy the bacillus [12]. Moreover, the LL form shows a predominance of M2 macrophages that induce the production of interleukin (IL)-10, transforming growth factor (TGF)-β, fibroblast growth factor (FGF)-β, arginase 1, CD209, CD163, and IDO, which contribute to the immunosuppressive response as well as tissue repair [13, 14].

There is growing evidence pointing to a new subpopulation of macrophages known as M4, which arise from M0 macrophages that change their behavior in the presence of CXCL4 to differentiate into M4 macrophages and produce CD68, IL-6, TNF-α, MRP8, matrix metalloproteinase (MMP)7, and MMP12 [15–17]. The first study on M4 macrophages showed their predominance in atherosclerotic lesions, which increase the expression of receptors for low-density lipoprotein (LDL), thereby provoking the accumulation of oxidized LDL in phagocytes and ultimately causing the development of atheroma plaques and oxidative lesions [18].

Although it is known that macrophages are the main cells participating in the host immune response against *M. leprae* infection, the behavior of this new M4 subtype of macrophages and their potential influence on the development of the in-situ immune response in the leprosy spectrum remain unknown. Such information could help broaden the discussion about the immunopathogenesis of the disease. Therefore, we investigated the responses of M4 macrophages in the polar forms of leprosy.

## Materials and methods

### Study design and participants

Biopsy samples of 33 untreated patients (25 men and 8 women) at the Center of Tropical Medicine, Federal University of Para, and Dermatology Department of State University of Para with a confirmed diagnosis of leprosy that was made according to the classification of Ridley-Joplin were analyzed in this study; 18 patients had tuberculoid leprosy (TT) and 15 had lepromatous leprosy (LL). All patients were from the state of Para, Brazil, and their mean age was 25.6 years.

### Histopathology and immunohistochemistry

For histopathological analysis, 5-μm thick slices were prepared from tissue biopsies, embedded in paraffin, and stained with hematoxylin and eosin.

Tissue-specific staining was achieved through immunohistochemistry using the biotin-streptavidin-peroxidase method with antibodies against CD68 (CM033C; Biocare Medical, Pacheco/CA, USA), MRP8 (ab92331; Abcam, Cambridge/MA, USA), MMP7 (ab205525; Abcam, Cambridge/MA, USA), IL-6 (ab154367; Abcam, Cambridge/MA, USA), and TNF-α (ab6671; Abcam, Cambridge/MA, USA). First, the tissue samples were deparaffinized in xylene and hydrated in a decreasing alcohol series. Endogenous peroxidase was blocked by incubating the sections in 3% hydrogen peroxide for 45 min. For antigen retrieval, the sections were incubated in citrate buffer (pH 6.0) at 90 °C for 20 min. Next, non-specific proteins were blocked by incubating the sections in 10% skim milk for 30 min. The histological sections were then incubated with the primary antibodies diluted in 1% bovine serum albumin for 14 h. Then, the slides were immersed in 1× phosphate-buffered saline (PBS) and incubated with the secondary biotinylated antibody [labeled streptavidin biotin (LSAB), Dako Cytomation] in an oven for 30 min at 37 °C. The slides were again immersed in 1× PBS and incubated with streptavidin peroxidase (LSAB) for 30 min at 37 °C. The reaction was developed with the addition of 0.03% diaminobenzidine plus 3% hydrogen peroxide as the chromogen solution. The slides were stained with Harris hematoxylin for 1 min, dehydrated in an increasing alcohol series, and cleared in xylene. CD68 and MRP8 double staining was conducted on the same histological sections, using streptavidin alkaline phosphatase and diaminobenzidine and as a chromogenic substrate (yielding a pink reaction product), according to the protocol described by Azevedo et al. [19].

### Quantitative analysis and photodocumentation

The immunohistochemical staining-positive areas were quantified using as a criterion of positivity the brownish deposit to coincide with macrophage morphology in the granulomatous infiltrate in the dermis. Immunostaining was quantified in five randomly selected fields that were visualized under an Axio Imager Z1 microscope (model 4,560,006; Zeiss) at a magnification of 400× using a 0.0625-mm^2^ grid with 10 × 10 subdivisions in the granulomatous inflammatory infiltrate, according to a previously described protocol [20–22].

### Statistical analysis

Data were stored in electronic spreadsheets of the Excel 2007 program. Statistical analysis was performed using GraphPad Prism V.5.0. In univariate analysis, frequencies and measures of central tendency and dispersion were obtained. The Mann-Whitney t-test and Spearman correlation test were applied to test the hypotheses. A threshold significance level of 5% (*p* ≤ 0.05) was adopted for all tests.

## Results

### Characteristics of the study subjects

The patients had altered tactile and thermal, and/or painful sensations on dermatoneurological examination. Patients with the TT form had cutaneous lesions consisting of erythematous or erythematous-hypochromic plaques with sharp edges and most anesthetic. Patients with the LL form had hypochromic spots and diffuse erythematous plaques and erythematous-violet or nodules that were infiltrated, bright, and sometimes coalescing. Histopathologically, the TT form was characterized by the presence of granulomas constituted of groups of epithelioid cells and sometimes surrounded by a dense or mild lymphocytic halo, with bacillus-negative status. In the LL form, we observed granulomatous infiltrate consisting of histiocytes and plasma cells, extending along the entire upper dermis and surrounding the nerves and blood vessels, which could involve the deep dermis to the hypodermis and had bacillus positive status.

### Immunohistochemical characterization of M4 macrophages

In tissue immunostaining, M4 macrophages were visible as depositions of brown-stained material in the cytoplasm or around cells, contrasting with the immunostaining-negative blue background (hematoxylin counterstaining). The presence of brown-stained areas coinciding with cell morphology was defined as a positive event. In the double staining experiment, brown-stained areas associated with pink-stained areas were areas positive for CD68 and MRP8. These criteria were adopted to minimize the counting of nonspecific staining, resulting in more accurate quantification.

**Fig. 1:**
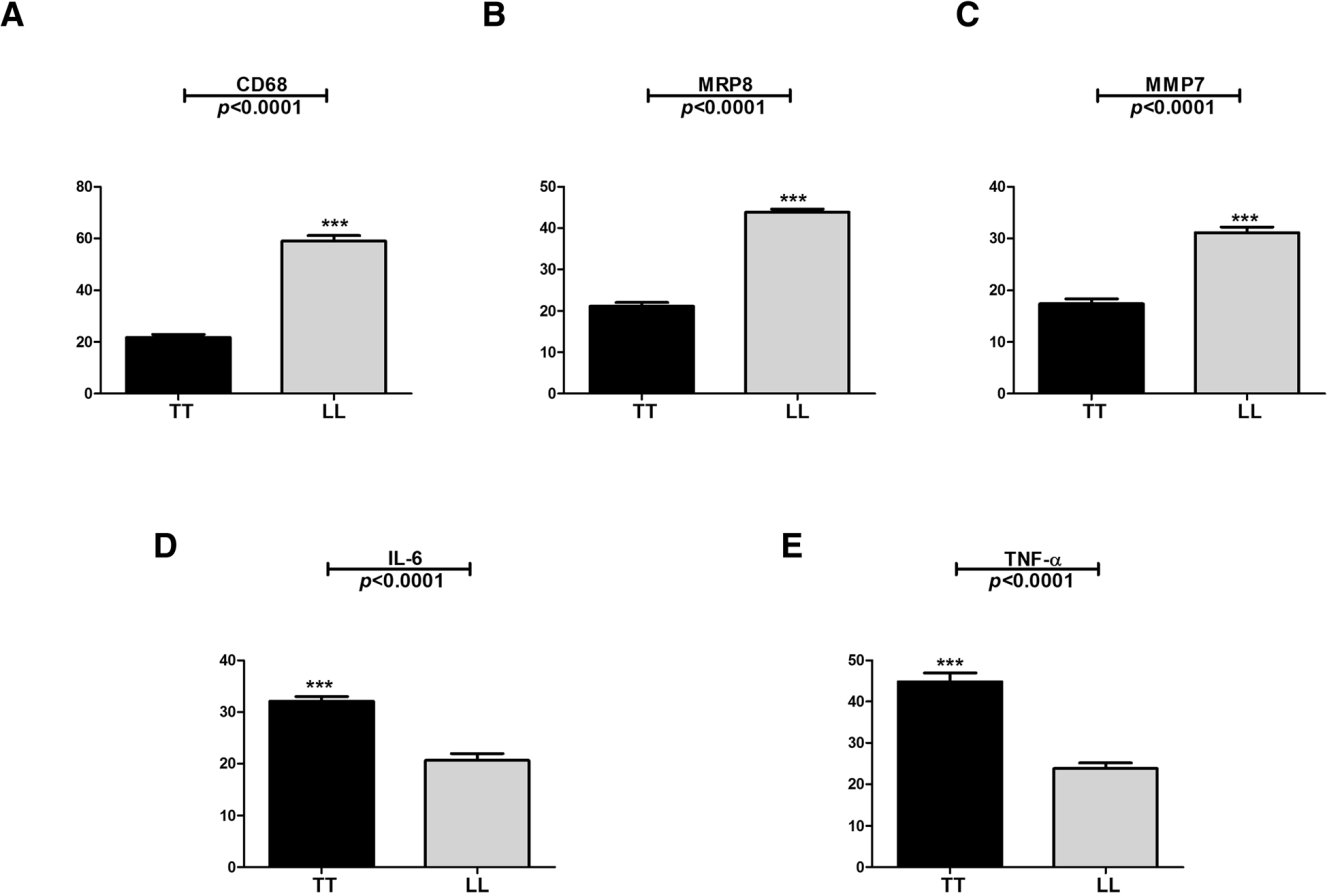
Quantitative analysis for the immunostaining of CD68 (**a**), MRP8 (**b**), MMP7 (C) and IL-6 (**d**) and TNF-α (**e**) in TT and LL forms of leprosy

**Fig. 2:**
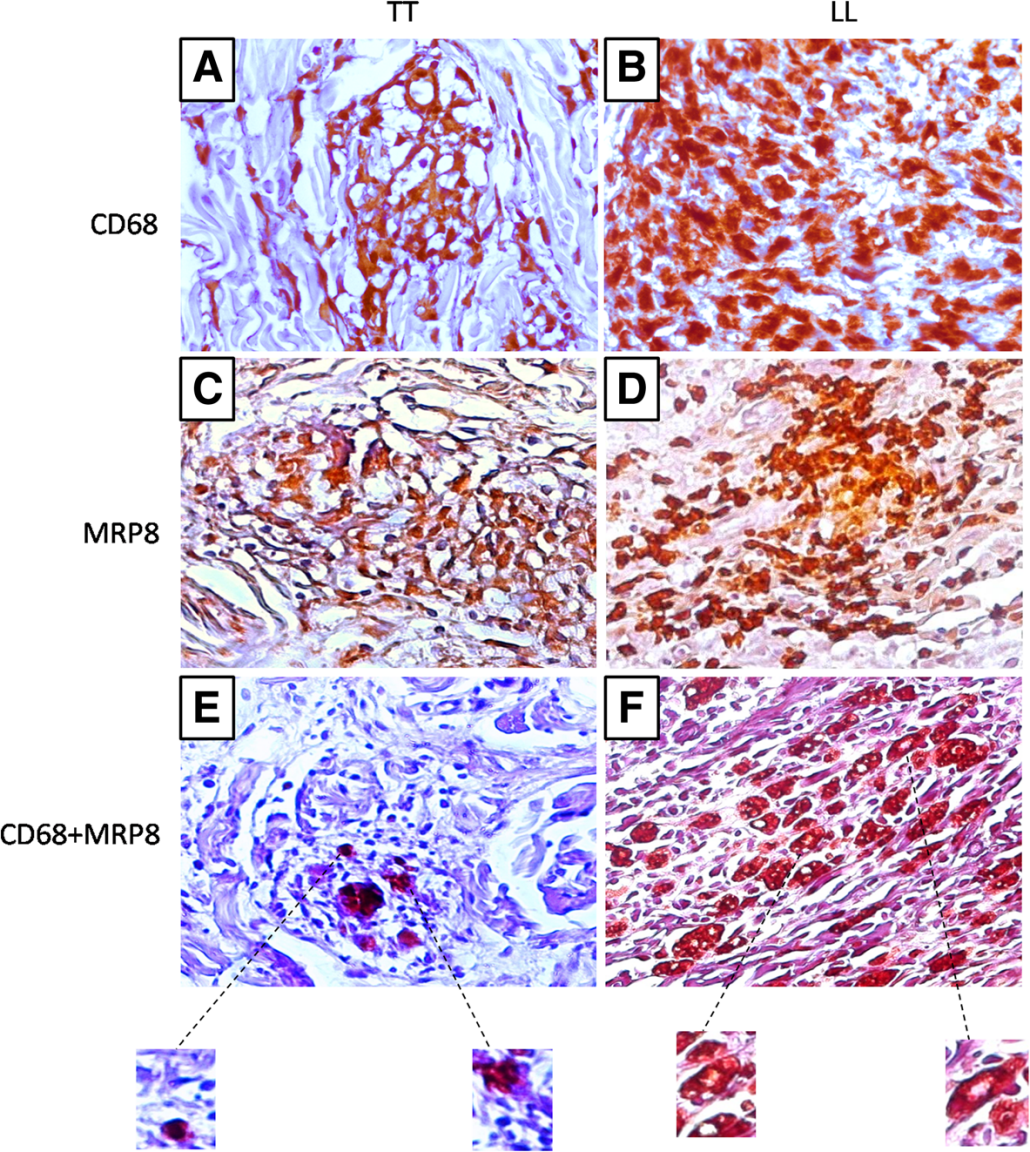
Positive immunohistochemistry for CD68 (**a**: TT, **b**: LL), MRP8 (**c**: TT, **d**: LL) and double labeling for CD68/MRP8 (**e**: TT, **f**: LL) in TT and LL forms of leprosy

**Fig. 3:**
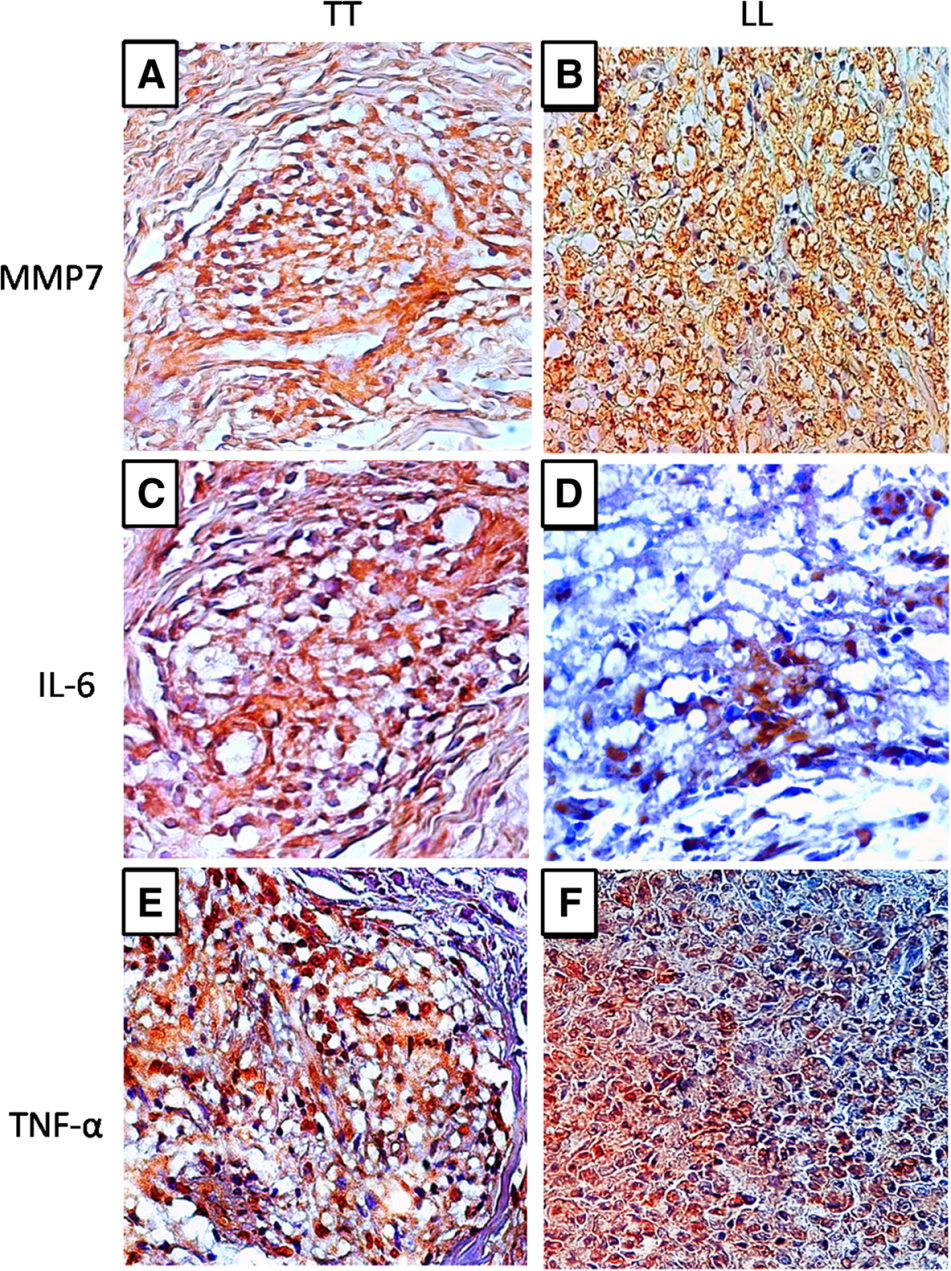
Positive immunohistochemistry for MMP7 (**a**: TT, **b**: LL), IL-6 (**c**:TT, **d**: LL) and TNF-α (**e**: TT, **f**: LL) in TT and LL forms of leprosy

Immunostaining for CD68 differed between the groups studied, with a significantly (*p* < 0.0001) lower median number of stained cells observed in the TT group (22.00 ± 3.55 cells/field) than in the LL group (61.00 ± 6.58 cells/field) (Figs. 1a, 2a and b). The median immuno-expression of MRP8 (Figs. 1b, 2c and d) and MMP7 (Figs. 1c, 3a and b) was also significantly (both *p* < 0.0001) lower in the TT group (MRP8: 21.50 ± 2.82 cells/field, MMP7: 17.00 ± 2.98 cells/field) than in the LL group (MRP8: 44.50 ± 2.57 cells/field, MMP7: 31.50 ± 3.44 cells/field). However, the immuno-expression of IL-6 and TNF-α was significantly (both *p* < 0.0001) higher in the TT group (IL-6: 32.00 ± 2.76 cells/field, TNF-α: 43.00 ± 6.81 cells/ field) than in the LL group (IL-6: 21.00 ± 4.30 cells/field, TNF-α: 24.00 ± 4.21 cells/field) (Figs. 1d, e, 3c-f). The double positive labeling for CD68 and MRP8 confirmed the presence of M4 macrophages in leprosy skin lesions (Figs. 2e and f).

Linear correlation analysis of immuno-expression in lesions of the TT and LL patients showed several positive associations, highlighting synergistic effects among CD68, MRP8, and MMP7 in the TT and LL forms (Table 1).

**Table 1:** Linear correlation analysis between markers that characterize the response of M4 macrophages in polar forms of leprosy

Correlation	TT	LL
CD68 x MRP8	*r* = 0.7796 *p* = 0.0078**	*r* = 0.6821 *p* = 0.0298*
CD68 x MMP7	*r* = 0.6895 *p* = 0.0312*	*r* = 0.7222 *p* = 0.0183*
CD68 x IL-6	*r* = 0.6364 *p* = 0.0479*	*r* = 0.0615 *p* = 0.8993
CD68 x TNF-α	*r* = 0.6771 *p* = 0.0315*	*r* = 0.7477 *p* = 0.0129*
MRP8 x MMP7	r = 0.6895 p = 0.0312*	*r* = 0.6604 *p* = 0.0377*
MRP8 x IL-6	*r* = 0.4458 *p* = 0.1966	*r* = 0.2609 *p* = 0.4666
MRP8 x TNF-α	*r* = 0.2883 *p* = 0.4191	*r* = 0.2050 *p* = 0.5700
MMP7 x IL-6	*r* = 0.1734 p = 0. 6319	*r* = 0.1486 *p* = 0.6820
MMP7 x TNF-α	*r* = 0.4939 *p* = 0.1468	*r* = 0.2724 *p* = 0.4463
TNF-α x IL-6	*r* = 0.4644 *p* = 0.1763	*r* = −0.1111 *p* = 0.7599

## Discussion

Leprosy is an intriguing immunologic complex disease in which *M. leprae* causes granulomatous lesions and demyelination in the peripheral nerves [23, 24]. Leprosy is considered a spectral disease, with clinical and histopathological changes showing strong relationships with the pattern of the immune response triggered in the host [6, 25].

Macrophages belong to a select group of cells that differentiate, go through phenotypic modification, and participate in the microbicidal response in the activation of the classical pathway by M1 macrophages or in tissue repair in response to the action of M2 macrophages [26, 27]. Recently, the involvement of M4 macrophages in the pathogenesis of atherosclerosis has been recognized; however, the role of this new subtype in leprosy has not yet been investigated [28, 29].

The results obtained in the present study suggest that M4 macrophages have characteristics that imply they are probably ineffective in the microbicidal response to *M. leprae*, thus contributing to the development of clinical forms with more lesions and enhanced bacillary proliferation, as observed in the LL form. Within this context, the immunosuppressive behavior of M4 macrophages in inhibiting the microbicidal response [30, 31] strongly suggests a possible role in mediating the immune response in the LL form of disease.

The first report of the emergence of M4 macrophages showed that phagocytosis might be completely suppressed in these macrophages, which is likely directly related to the low expression of CD163, a scavenger receptor that recognizes hemoglobin/haptoglobin complexes [32]. In the LL form, this problematic characteristic of M4 macrophages might be crucial for maintaining the survival of the bacillus in the phagocytes owing to pathogen-triggered immune evasion. Therefore, the response of M4 macrophages as well as that of M2 macrophages suggests that the immunosuppressive environment established in the LL form of leprosy can restrict the microbicidal response to facilitate bacillus proliferation, resulting in more numerous lesions [13, 14].

Considering the cellular infiltrates, it is worth mentioning that the predominance of M4 macrophages in diseases such as atherosclerosis demonstrates that cells change their behavior favoring the appearance of foam cells and the development of an oxidative stress response inducing chemokine production and monocyte recruitment, thereby facilitating the accumulation of macrophages that express large amounts of LDL receptors [33, 34]. One of the greatest challenges associated with immunopathological studies of the LL form lies in understanding the activity of macrophages and the differentiation mechanisms that influence their morphological patterns [35]. Through the numerous changes that occur in the tissue environment, Virchow’s cells emerge as part of the adaptive process, which demonstrates that in the chronicity of the inflammatory response, macrophages lose the ability to destroy the bacillus, and lipid degeneration caused by the oxidative stress favors the appearance of foamy macrophages with vacuoles containing large numbers of bacilli [35–37]. Through the immunolabeling of markers that characterize the response of M4 macrophages (CD68, S100A8, and MMP7), we observed a statistically significant difference in M4 macrophages in the LL form compared to the TT form.

Moreover, correlation analysis revealed an association between the expression of CD68, S100A8, and MMP7, which probably results in increased cellular activity in the polar disease forms. Of note, in the LL form, the expression of CD68, S100A8, and MMP7 was predominant in the inflammatory infiltrate composed of numerous foamy macrophages. The predominance of CD68 in the LL form of leprosy has been previously reported. Furthermore, the CD68 level is positively correlated with the production of iNOS in the microbicidal response in TT form of leprosy, which is one of the main enzymes that induce the production of NO and free radicals [38].

MRP8 (also known as S100A8 or calgranulin A) has been linked to numerous regulatory functions that modulate cell differentiation as well as phagocyte recruitment and activity [39, 40]. MRP8 exhibits ambiguous behavior in response to *Mycobacterium tuberculosis* infection*.* In macrophages infected with *M. tuberculosis*, MRP8 formed a complex with MRP14 that facilitated bacillus survival [41]. In contrast, other studies have shown that macrophages infected with *M. tuberculosis* or *M. leprae* had increased MRP8 activity of the phagolysosome, mainly due to the response of IL-22 [42, 43].

MMP7 (also known as matrilisin) is a zinc- and calcium-dependent endopeptidase that degrades the extracellular matrix and regulates various cellular processes, including cellular proliferation, tissue remodeling, the inflammatory response, and apoptosis [44, 45]. In an attempt to control the environment of tissue stress, increased MMP7 expression may mediate the tissue repair response by acting together with other cytokines, such as TGF-β and NGF, to promote tissue regeneration, and thus avoid the development of multiple lesions that are characteristic of LL clinical form [46, 47].

Finally, we investigated the expression levels of IL-6 and TNF-α in the TT and LL forms of the disease, and we found that both IL-6 and TNF-α are increased in the TT form. Classically, IL-6 and TNF-α are considered to be cytokines that are strongly associated with the development of the M1 macrophage response and induction of the microbicidal response. In the TT form, these cytokines also participate in the responses of the lymphocytes Th1, Th17, and Th22, thereby aggravating the tissue damage [43, 46, 47].

## Conclusion

Our study demonstrated that the presence of M4 macrophages in the LL skin lesions may be involved in an infective immune response and consequently the survival of *M. leprae.* Previous findings on the pathogenesis of atherosclerosis and the formation of vacuolated macrophages morphologically similar to Virchow’s cells support our immunohistopathological findings in the LL form of leprosy. Our data also suggest that these cells can induce the establishment of a regenerative environment and remodeling of the extracellular matrix, which are important for the pathogen–host interaction during infection by *M. leprae*. Further studies in experimental models are needed to elucidate the detailed mechanisms underlying the roles of M4 macrophages in the pathogenesis of leprosy lesions and provide further insights into the disease spectrum.
